# A novel quantification-driven proteomic strategy identifies an endogenous peptide of pleiotrophin as a new biomarker of Alzheimer’s disease

**DOI:** 10.1038/s41598-017-13831-0

**Published:** 2017-10-17

**Authors:** Tobias Skillbäck, Niklas Mattsson, Karl Hansson, Ekaterina Mirgorodskaya, Rahil Dahlén, Wiesje van der Flier, Philip Scheltens, Floor Duits, Oskar Hansson, Charlotte Teunissen, Kaj Blennow, Henrik Zetterberg, Johan Gobom

**Affiliations:** 1 Institute of Neuroscience and Physiology, Department of Neurochemistry, the Sahlgrenska Academy at the University of Gothenburg, Mölndal, Sweden; 2 Clinical Neurochemistry Laboratory, Sahlgrenska University Hospital, Mölndal, Sweden; 3 Clinical Memory Research Unit, Department of Clinical Sciences Malmö, Lund University, Malmö, Sweden; 4 Department of Neurology, Skåne University Hospital, Lund, Sweden; 5 Proteomics Core Facility, Sahlgrenska Academy, University of Gothenburg, Gothenburg, Sweden; 6 Alzheimer Centre, Amsterdam Neuroscience, VU University Medical Center, Amsterdam, The Netherlands; 7 Department of Molecular Neuroscience, UCL Institute of Neurology, Queen Square, London, UK

## Abstract

We present a new, quantification-driven proteomic approach to identifying biomarkers. In contrast to the identification-driven approach, limited in scope to peptides that are identified by database searching in the first step, all MS data are considered to select biomarker candidates. The endopeptidome of cerebrospinal fluid from 40 Alzheimer’s disease (AD) patients, 40 subjects with mild cognitive impairment, and 40 controls with subjective cognitive decline was analyzed using multiplex isobaric labeling. Spectral clustering was used to match MS/MS spectra. The top biomarker candidate cluster (215% higher in AD compared to controls, area under ROC curve = 0.96) was identified as a fragment of pleiotrophin located near the protein’s C-terminus. Analysis of another cohort (n = 60 over four clinical groups) verified that the biomarker was increased in AD patients while no change in controls, Parkinson’s disease or progressive supranuclear palsy was observed. The identification of the novel biomarker pleiotrophin 151–166 demonstrates that our quantification-driven proteomic approach is a promising method for biomarker discovery, which may be universally applicable in clinical proteomics.

## Introduction

The development of proteomic techniques based on mass spectrometry has enabled an explorative approach to identifying new biomarkers among large numbers of proteins or peptides in clinical samples and detecting disease-associated differences in their abundances in study cohorts^1,2^. In medical research into neurodegenerative disorders, such as Alzheimer’s disease (AD), biomarkers in cerebrospinal fluid (CSF) play a central role for the understanding of pathologic mechanisms and as an aid in drug development^3^. In clinical settings they are gaining importance as a support in diagnostics and as indicators of disease progress^4^.

Disease heterogeneity and experimental variation often necessitates the analysis of large study groups in order to obtain sufficient statistical power to single out biomarker candidates among hundreds or thousands of identified proteins. While study size was previously limited by the lengthy liquid chromatography-mass spectrometry (LC-MS) analyses required in discovery proteomics workflows, performing large studies comprising hundreds of participants is now feasible through the development of timesaving multiplex isobaric labeling techniques, such as the tandem mass tag (TMT) approach^5^. At present, the TMT technique accommodates 10-plexing, i.e., up to 10 individual samples can be combined in one TMT set and analyzed in one LC-MS run^6^.

Currently, an identification-driven strategy is most commonly used for proteomic data analysis, including in studies based on isobaric labeling. According to this strategy, peptide identification is performed as the first step by searching fragment ion (MS/MS) spectra against protein sequence databases^7^. A limitation of this approach for biomarker discovery is that only spectra that result in peptide identification are further evaluated, while unidentified spectra, generally constituting the majority in proteomic LC-MS data sets, are discarded. There are several potential reasons why peptides elude identification: the sequence may be a variant not entered into the sequence database, the peptide may carry an unexpected post translational modification, or its MS/MS fragmentation characteristics may be unsuitable for identification. While not permitting immediate peptide identification, these spectra may nevertheless contain quantitative information on biomarker candidates, which is overlooked because their identity could not be established in the first step of the analysis.

To address this limitation, we developed a new quantification-driven proteomic approach that uses spectral clustering^8^ (instead of identified peptide sequence) to match MS/MS spectra representing the same peptide in isobaric labeling proteomic LC-MS data sets. All clustered data are evaluated quantitatively for their ability to separate the study groups, identifying biomarker clusters that can be subsequently identified in targeted follow-up experiments.

Using this approach, we compared the CSF endopeptidomes (naturally occurring peptides, i.e., not obtained by *in vitro* proteolysis) of 40 AD patients with 40 non-demented controls to identify biomarker candidates of AD. Also included in the study were 40 patients with mild cognitive imapairment (MCI)^9^. While not a separate disease entity, MCI is characerized by a set of measurable symptoms of cognitive decline that often preceeds AD but do not meet the clinical criteria of AD or other dementia disorders. An important goal in AD research is to identify those MCI patients that will progress to AD, at an early stage.

The identification of an endogenous peptide of pleiotrophin as a new AD biomarker demonstrates that the quantification-driven approach enables identification of new biomarkers that lie outside the boundaries of identification-driven analysis.

## Results

**Figure 1:**
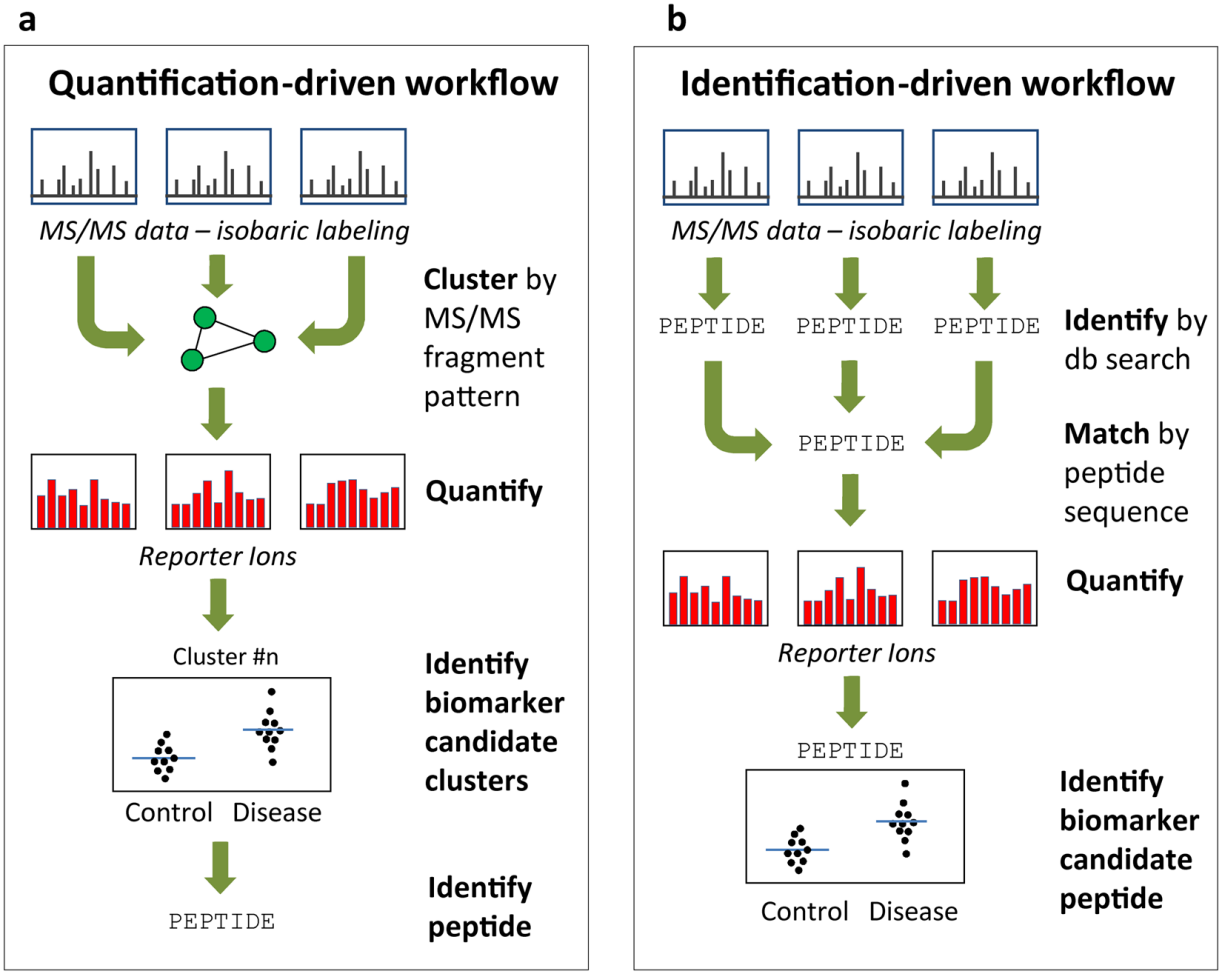
Quantification-driven versus identification-driven proteomics. In the quantification-driven workflow (**a**), all MS/MS data, including data from different TMT sets and technical replicates, are clustered using an algorithm that matches spectra based on precursor ion *m/z*, charge state, and similarity of fragment ion pattern. For each cluster, the relative peptide abundance in the study participants, determined by the TMT reporter ion data, is used to single out biomarker candidate clusters, which are subsequently identified in targeted follow-up experiments. In contrast, in the identification-driven workflow (**b**), peptide identification is performed as the first step of data processing and the identified peptide sequences are used to match spectra for quantification. Consequently, only spectra that yield a positive identification can be included for biomarker evaluation.

A quantification-driven proteomic workflow (Fig. 1a) was designed in which all acquired MS/MS data, including data from the different TMT sets and from technical replicates, are clustered based on precursor *m/z*, charge, and similarity of fragment ion pattern. Within each cluster, TMT reporter ion ratios are compared between study groups to identify candidate biomarker clusters. The peptide sequences of the candidates are then determined in targeted follow-up experiments. In the identification-driven workflow (Fig. 1b), which is commonly used in proteomics, identification is performed as the first step of the analysis and only those spectra that yield positive identification are used for quantification. We hypothesized that performing quantitative analysis of all spectra would enable detection of biomarker candidates among unidentified MS data, and that these candidates can then be identified by tailoring the follow-up experiments to the selected analyte.

### Biomarker candidate cluster discovery

The quantification-driven workflow was used to analyze the CSF endopeptidome of a cohort consisting of 120 participants consisting of 40 patients with a clinical diagnosis of probable AD, 40 patients with MCI, and 40 controls (C) with subjective cognitive decline but not meeting the criteria for MCI or other dementia diseases (Supplementary Table S1). At follow-up, 14 of the MCI patients had progressed to AD dementia (MCI-AD) while 23 remained stable MCI (MCI-S). Three MCI patients were later diagnosed with a different disease (MCI-OD). For statistical analysis to identify biomarker candidates we selected the subset of probable AD patients that met the IWG-2 criteria^10^, based on clinical assessment and CSF biomarker data (AD-IWG-2), while seven patients (Prob. AD) were excluded.

**Figure 2:**
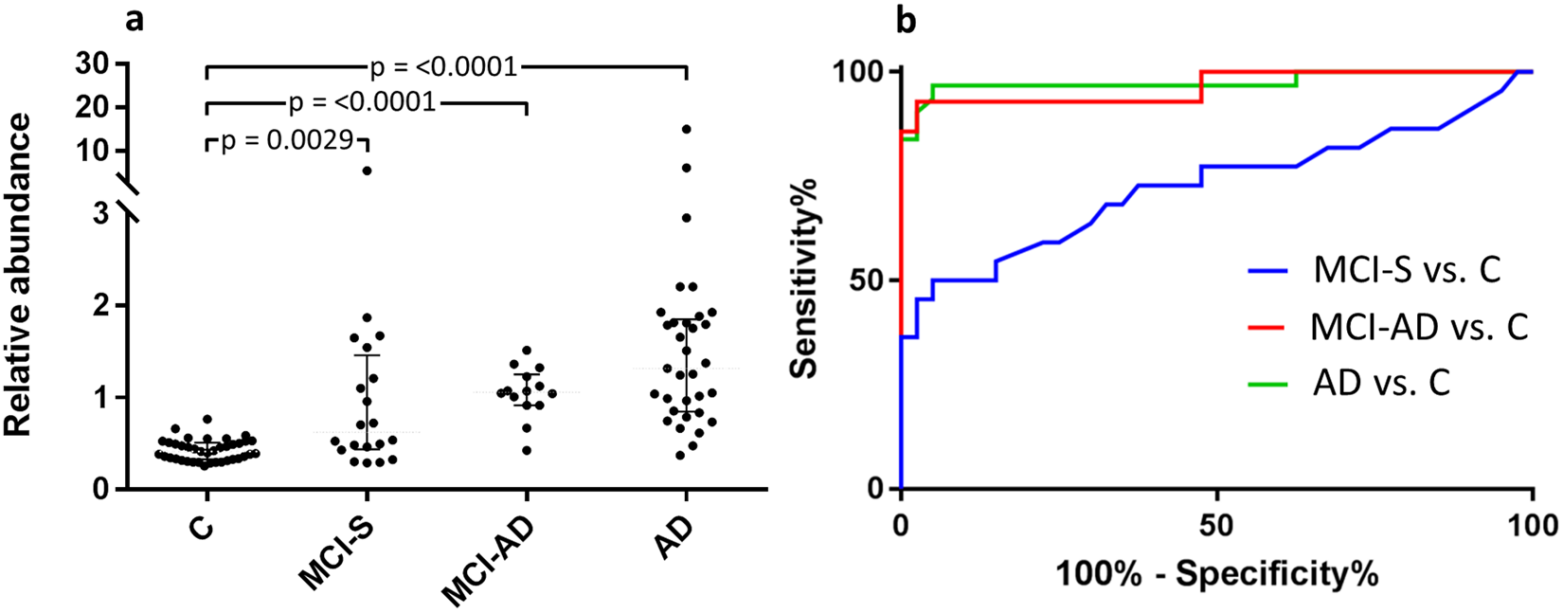
Top biomarker candidate cluster #7367. (**a**) Scatter plot of the relative abundance over the different patient groups. The cluster was increased by 222% in AD versus C, by 153% in MCI-AD versus C and by 35% in MCI-S versus C. The p-values were obtained by Mann-Whitney test. (**b**) ROC curve analysis. The cluster had an AUC of 0.96 for separating AD versus C, an AUC of 0.96 for MCI-AD versus C and an AUC of 0.72 for MCI-S versus C.

The CSF samples were labeled using TMT10-plex reagents and endogenous peptides, isolated by molecular weight ultrafiltration, were analyzed by LC-MS in quadruplicate using identical data-dependent acquisition settings, resulting in a data set comprising 1,200,468 MS/MS spectra. The spectra were divided into 220,869 clusters by the clustering algorithm. Out of these, 27,065 clusters that had a quantifiable signal in > 50% of the study subjects were evaluated using ROC curve analysis, comparing the AD-IWG-2 group with the control group. The clusters were ranked as biomarker candidates according to decreaseing area under the ROC curve (AUC). The top 20 clusters are listed in Table 1. The top ranking biomarker candidate was cluster #7367, represented by a +5 charge signal with an apparent *m/z* 794.115 (monoisotopic). A scatter plot of the TMT ratios for the cluster over the patient groups is shown in Fig. 2. Having an AUC of 96% for separating AD from C, a 215% higher abundance in AD compared to C, and being detected in 119 out of 120 participants made this cluster particularly interesting as a biomarker candidate and it was thus targeted for identification.

**Table 1:** Top 20 biomarker candidate clusters.

Cluster #	Cluster size (n)	m/z	Chg.	Cluster similarity (p-value)	Subjects (n)	Median change			AUC (C - AD)	AA Sequence	Protein name
						AD - C	MCI-AD - C	MCI-S - C			
7367	39	794.115	5	0.073	119	215%**	153%**	35%*	0.96*		
69078	18	664.799	2	0.000	94	18%**	15%**	0%	0.92*		
32527	11	777.426	3	0.001	68	13%**	8%*	8%	0.92*		
78065	41	748.382	2	0.001	120	38%**	20%**	9%*	0.91*	aDSGSSEEkQ	osteopontin
4243	10	696.457	5	0.000	59	103%*	67%**	59%**	0.91*		
82223	22	794.885	2	0.000	111	28%**	22%**	11%**	0.90*	tVASHTSDSDVPSG	clusterin
34935	10	810.044	3	0.001	58	16%**	16%*	-1%	0.9*		
9084	13	836.597	5	0.000	86	47%**	35%**	27%*	0.87*		
36033	34	823.45	3	0.006	120	18%**	13%*	3%*	0.87*	kVEQAVETEPEPELRQQ	ApoE
61266	14	1380.71	3	0.000	70	−20%**	−7%	−10%*	0.86*		
1889	53	427.579	3	0.014	120	15%**	8%*	6%	0.86*		
33238	10	787.612	3	0.001	69	19%**	34%**	15%*	0.85*		
25327	14	684.044	3	0.003	60	9%**	9%*	10%*	0.85*		
13290	25	840.651	4	0.038	111	17%**	16%**	7%	0.85*	gGSLPSEEkGHPQEESEESNVSMASLGE	secretogranin-1
10464	9	786.901	4	0.002	59	22%**	4%	8%	0.85		
71623	10	685.336	2	0.000	61	13%**	7%*	7%*	0.83*		
12243	10	911.104	5	0.000	70	−28%**	−7%	6%	0.82		
87816	44	848.511	2	0.002	69	16%**	24%**	6%	0.8*		
6405	11	690.794	4	0.000	60	13%*	−2%	−1%	0.79*		
53204	8	1088.54	3	0.000	60	4%*	16%*	8%	0.79*		

**Indicates statistical significance (p < 0.001), *Indicates statistical significance (p < 0.05). Top 20 spectral clusters sorted according to descending Area Under ROC curve in AD vs C. Columns (left to right) are: cluster id number; number of spectra included in the cluster; cluster average mass-to-charge ratio (*m/z*); charge; average p value of cluster similarity as calculated by the MS Cluster algortihm; the number of study subjects in which the cluster was quantified; median relative change in abundance between AD and C, between MCI-AD and C, and between MCI-S and C; area under curve from ROC curve analysis of C vs. AD; amino acid sequence of peptides identified by database searching; protein name.

All MS/MS spectra were submitted to peptide identification using the software programs Mascot (Matrixscience, UK) and PEAKS (BSI, Canada). The Mascot search identified peptides in 2,445 of the 27,065 clusters and PEAKS 6,585. Among the top 20 clusters listed in Table 1, sixteen (80%) had no peptide PSM. The four identified proteins were clusterin, osteopontin, apolipoprotein E, and secretogranin 1. The entire cluster list, annotated with peptide identifications is shown in Supplementary Data set 1.

### Identification of biomarker candidate

**Figure 3:**
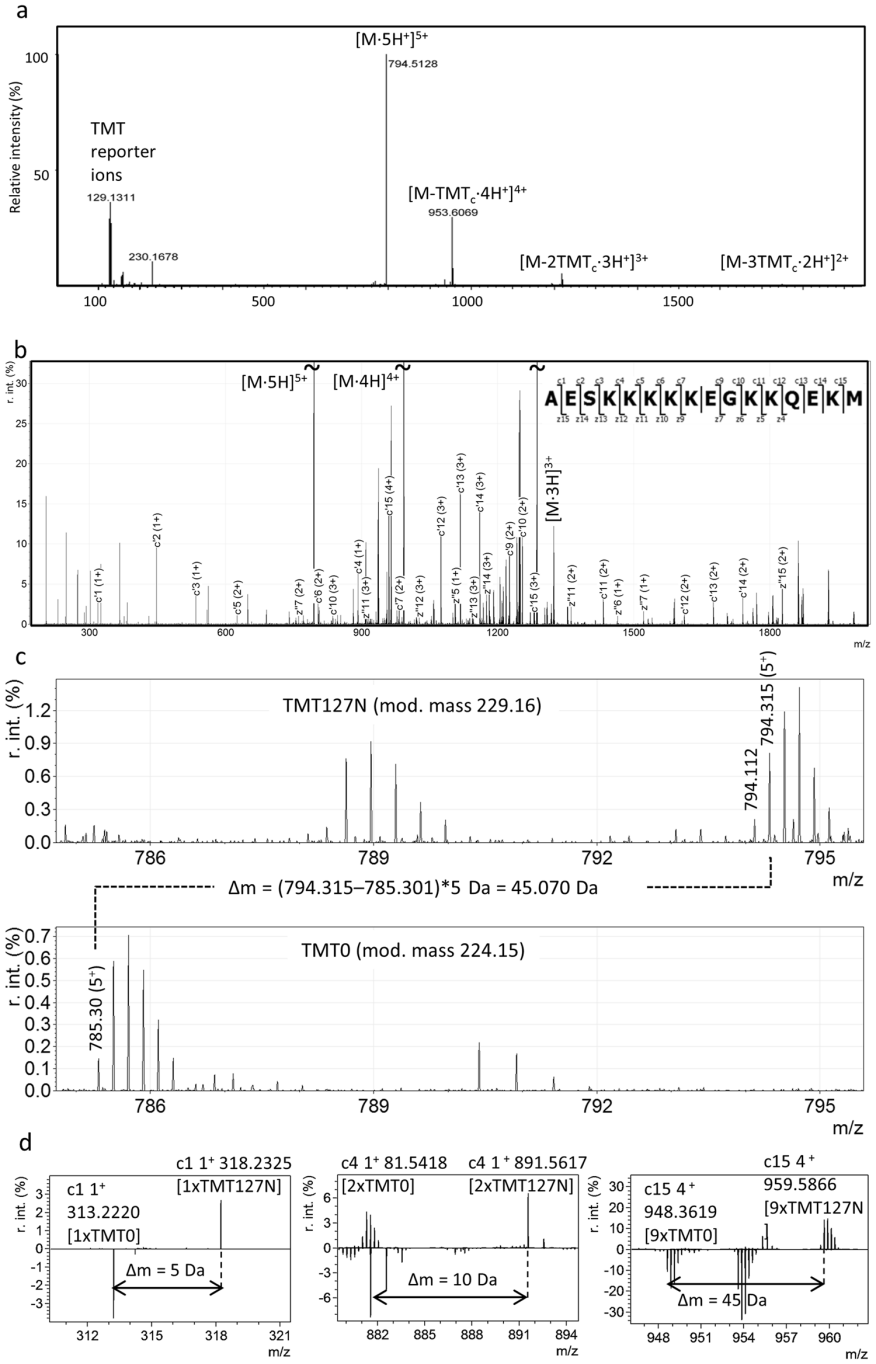
Identification of cluster #7367 as PTN 151–166 by *de novo* sequencing. (**a**) HCD MS/MS spectrum, featuring multiple TMT^c^ signals, indicating the presence of several lysine residues in the peptide. (**b**) ETchD MS/MS spectrum annotated with matching c- and z- ions (∆m < 0.01 Da) for PTN 151–166, identified by *de novo* sequencing. (**c**,**d**) Verification of PTN 151–166 by differential labeling of a CSF sample with TMT127N and TMT0: (**c**) The mass difference of 45 Da between the TMT127N labeled (top panel) and the TMT0 labeled (bottom panel) molecular ion verifies the presence of nine TMT labels on the peptide. (**d**) Comparison of the mass differnces for c-ions confirms the locations of labeled lysine residues in the sequence, e.g., c1 (left panel) has a ∆m = 5 Da corresponding to TMT-labeled N-terminus; c4 (middle panel) has ∆m = 10 Da corresponding to two TMT labels (N-terminus and Lys-4); c15 (right panel) has ∆m = 45 Da corresponding to nine TMT labels.

Besides the TMT reporter ions, the fragment ion signals in the higher-energy collisional dissociation (HCD) MS/MS spectrum of cluster #7367 consisted of a +4 charge signal of *m/*z 953.609, a +3 charge signal of *m/z* 1219.436, and +2 charge signal of *m/z* 1751.087, corresponding to consecutive losses of a 156.13 Da moiety along with reduction of the charge state by one per loss (Fig. 3a). This behavior is characteristic of TMT complement cluster (TMT^c^) ions^11^. Formed by cleavage of the amide bond of the TMT, the TMT^c^ contains the peptide and most of the mass-balancing part of the TMT. The high charge of the precursor ion and the observed TMT^c^ signals indicate multiple TMT labels, suggesting the presence of several Lys residues in the sequence. However, apart from these features, the MS/MS spectrum did not feature any fragmentation that could be used to identify the peptide sequence.

To identify cluster #7367, new peptide extract from a CSF pool was prepared, labeled with one of the TMT10-plex reagents and analyzed by LC-MS using fragmentation by electron-transfer and higher-energy collision dissociation (EThcD)^12^ (Fig. 3b). Charge state deconvolution and the Manual Auto *de novo* sequencing function in PEAKS Studio^7^ was used to compute sequence tags from the spectra, resulting in several tags containing multiple Lys residues labeled with TMT. The sequence AESKKKKK, present in all proposed tags, was searched against the UniRef100 database using MS BLAST (http://genetics.bwh.harvard.edu/msblast/)^13^, and was found to match a sequence stretch within the protein pleiotrophin (PTN). By repeating the EThcD experiment with CSF samples labeled with TMT0 (a form of the TMT reagent that does not incorporate heavy isotopes) it was possible to establish that the peptide carried nine TMT tags (Fig. 3c). Further, these experiments revealed that the monoisotopic *m/z* of the peptide was 794.31, not 794.11 as had previously been assigned. We conclude that the *m/z* 794.11 signal is likely to be caused by isotopic impurities that are present in the heavy TMT reagents, giving rise to signals of [M-1] Da. The peptide monoisotopic mass (3966.54 Da) matched amino acid 151–166 of PTN, with the sequence AESKKKKKEGKKQEKM (∆m = −0.01 Da). For complete TMT labeling this peptide would be expected to carry nine TMT tags (eight on the Lys residues plus one on the N-terminus), thus matching the predicted number. Annotating the EThcD spectrum with c- and z-ions calculated for PTN 151–166 produced a near-complete matching c-ion series up to c15, as well as a long stretch of matching z-ions (Fig. 3b). There were also several matching b-ions. All matching ions are listed in Supplementary Table S2. Taken together, these results provide conclusive evidence that biomarker cluster #7367 is pleiotrophin 151–166.

### Validation of of PTN 151–166 as a biomarker of AD

**Figure 4:**
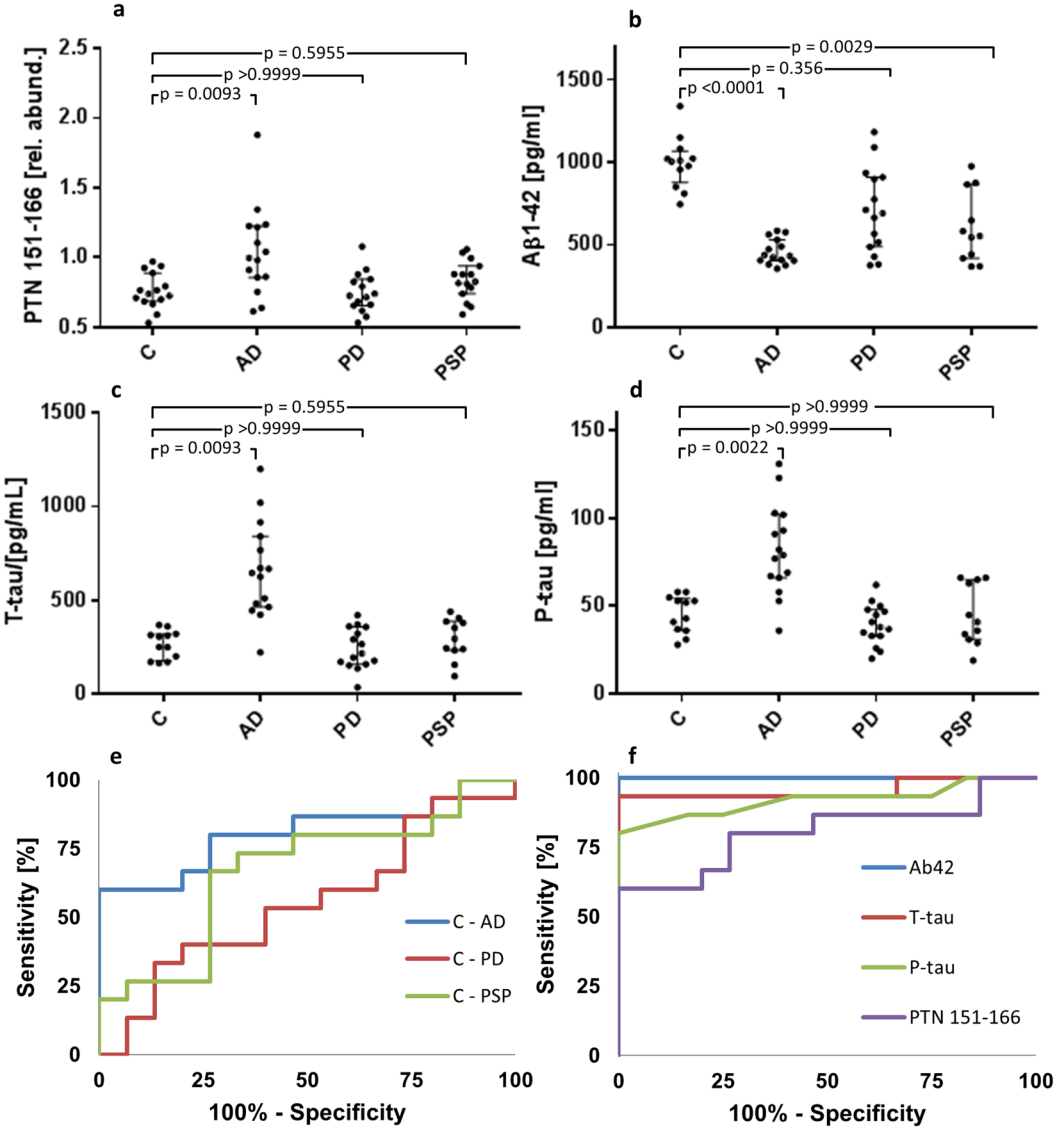
Validation of PTN 151–166 as a biomarker of AD. (**a**–**d**) Scatter plots of the relative abundance of (**a**) PTN 151–166, and the concentration of (**b**) β-amyloid 1–42, (**c**) t-tau and (**d**) P-tau across the different patient groups. (**e**,**f**) ROC curves of (**e**) PTN 151–166 for separating controls from AD (AUC = 0.80), PD (AUC = 0.55), and PSP (AUC = 0.67), respectively. (**f**) ROC curve for separating controls from AD of β-amyloid 1–42 (AUC = 1.0), t-tau (AUC = 0.96), P-tau (AUC = 0.92), and PTN 151–166 (AUC = 0.80).

To validate PTN 151–166 as a biomarker of AD, CSF of a second cohort (Supplementary Table S3) was analyzed that included 15 AD patients and 15 healthy controls (C), as well as 15 patients with Parkinson’s disease (PD) and 15 patients with progressive supranuclear palsy (PSP). This cohort was also analyzed using TMT10-plex labeling, using the same sample preparation and analytical procedure as was used for the discovery cohort, but instead of data-dependent acquisition, targeted MS2 of the +5-charge PTN 151–166 ion (m/z 794.115) was performed to ensure that MS2 spectra were recorded of the peptide in all samples. For those CSF samples of which sufficient volume remained, the core AD biomarkers Aβ42, T-Tau, and P-tau were analyzed using ELISA (n_C_ = 12, n_AD  _ = 15, n_PD_ = 15, n_PSP_ = 11). Figure 4a–d show scatter plots of the biomarkers across the disease groups. PTN 151–166 was significantly (p < 0.01) elevated in AD compared to controls (35% higher median) but not in the other disease groups. (Supplementary Table S4). Aβ42 was significantly decreased in the AD (−57%) and the PSP group (−45%), and T-tau and P-tau were significantly increased in the AD group (131% and 66%, respectively). The specificity of PTN 151–166 as a marker of AD was evaluated by ROC curve analysis (Fig. 4e). PTN151–166 separated AD from controls with an AUC of 0.80, while the corresponding AUCs for PD and PSP were 0.55and 0.67, respectively. ROC curve analysis was also used to compare the performance of PTN 151–166 (AUC = 0.80), Aβ42 (AUC = 1.0), T-tau (AUC = 0.96), and P-tau (AUC = 0.92) for separating AD from controls (Fig. 4f).

### Correlation of PTN 151–166 to other AD biomarkers

**Figure 5:**
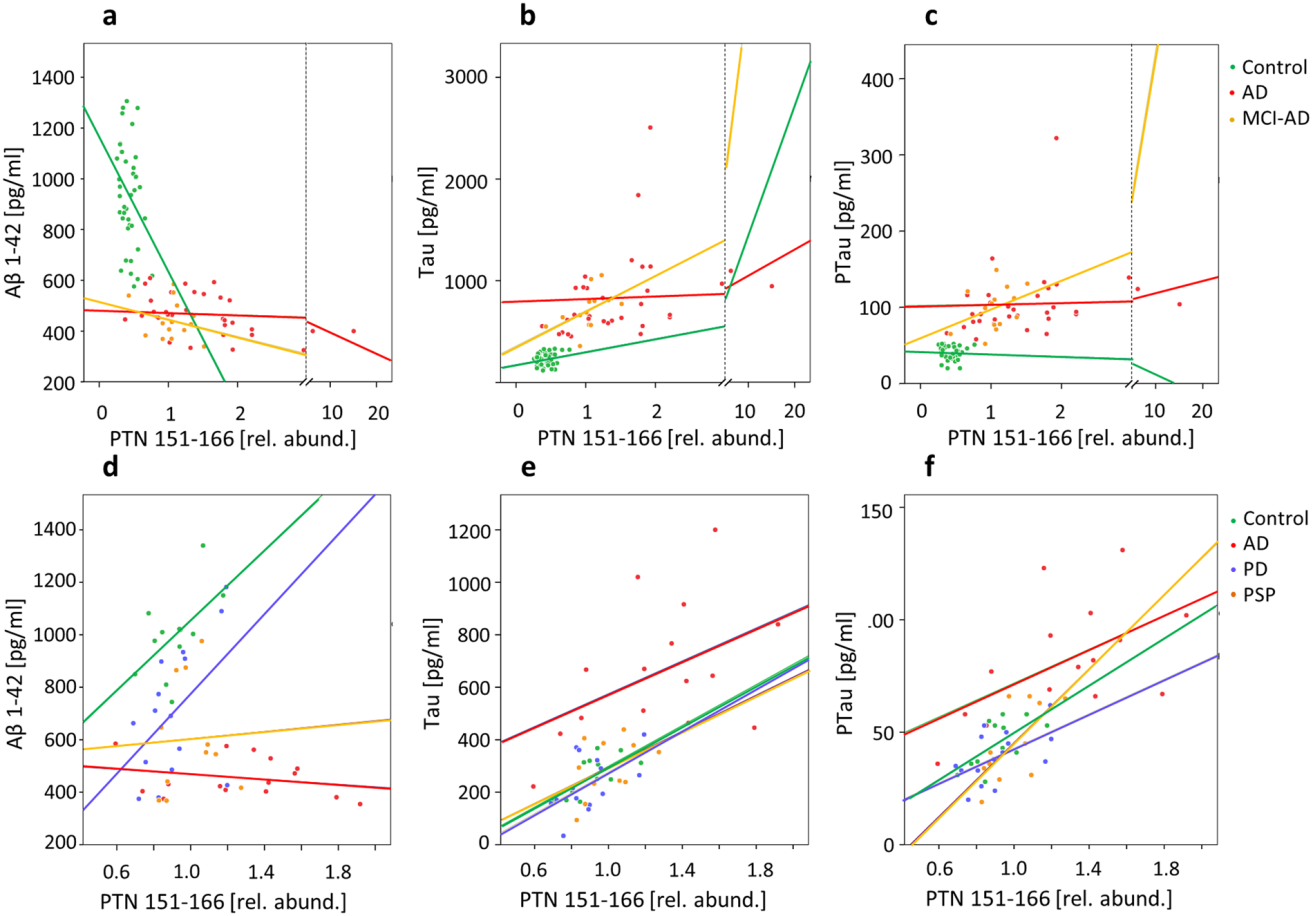
Correlations of PTN 151–166 to the core CSF AD biomarkers. Scatterplots of (**a**) Aβ42, (**b**) t-tau, and (**c**) P-tau versus PTN 151–166, in the C, AD (IWG-2), and MCI-AD groups of the discovery set.

The relative abundance of PTN 151–166 was compared to the core CSF biomarkers, β-amyloid 1–42 (Aβ42), the total concentration of microtubule-associated protein tau (t-tau) and its hyperphosphorylated form (P-tau). The correlations are shown in Fig. 5 and in Supplementary Table S5 (discovery set) and S6 (validation set). Among the AD patients in the discovery set, PTN 151–166 correlated with Aβ42 (r_s _= −0.505, p = 0.003), t-tau (r_s_= 0.570, p = 0.001) and P-tau (r_s_= 0.32, p = 0.011) but not in the control group. In the MCI-AD group PTN 151–166 correlated only with t-tau concentrations (r_s_= 0.53, p = 0.008), and in the MCI-S group PTN 151–166 correlated only with Aβ42 (r_s_= −0.55, p < 0.001), however the sample sizes in these groups were small (n = 14 and n = 22). No correlations were found in the control group. In the validation set, PTN 151–166 correlated with t-tau in the control group (r_s,_ = 0.629, p = 0.03) and in the PD group (r_s,_ = 0.515, p = 0.05), and with P-tau in the control group (r_s,_ = 0.656, p = 0.02), in the AD group (r_s_ = 0.514, p = 0.05), and in the PD group (r_s_ = 0.564, p = 0.03), and with t-tau only in the PD group (r_s_ = 0.515, p = 0.05), while it did not correlate to Aβ42 in any group.

## Discussion

### Biomarker identification using a quantification-driven proteomic approach

Our hypothesis that underlie the quantification-driven proteomic approach is that there are biomarker candidates in the analyzed samples that are not identified in a database search, but which can be found by mining the quantitative isobaric mass tag data, using spectral clustering, based on similarity of MS/MS peak pattern, to match together fragment ion spectra that represent the same peptides within and between LC-MS runs.

The observation that out of the 20 top candidates, shown in Table 1, only 20% were identified in the database search supports this hypothesis. The four identified clusters were all derived from proteins that have previously elicited interest in AD. Clusterin (or ApoJ) is an apolipoprotein that is genetically associated with AD and might play a pathogenic role in AD through effects on Aβ aggregation or other mechanisms, e.g. by immune system influence or by disrupting healing after neurodegeneration, and isoforms of clusterin has been suggested as a promising biomarkers in AD^14,15^. Osteopontin is a proinflammatory cytokine and CSF concentrations of osteopontin have been shown to be elevated in AD and MCI^16,17^, but it is not specific to AD since it is also associated with markers of increased axonal injury in frontotemporal lobe dementia^18^. ApoE transports lipids to neurons, and the ε4 allele of the *APOE* gene is the most prominent genetic factor for increased risk of AD^19,20^. Secretogranin-1, also known as chromogranin B, is a precursor molecule found in dense core vesicles and has been detected in neuritic plaques in AD^21,22^, and CSF levels of secretogranin-1-related peptides have been found to correlate with amyloid β and amyloid precursor protein (APP) peptides in CSF^23^. Endogenous peptides from secretogranin-1 have been identified in a previous study of AD and controls^24^. The fact that all four proteins from which the identified peptides (clusters) were derived have previously been proposed as biomarkers of AD supports the notion that the quantitative information of the clusters can be used to single out biomarker candidates.

Having singled out unidentified biomarker candidate clusters of interest, the next task to address was to determine why they were not identified and design experiments to identify them. Using higher-collisional energy dissociation (HCD); the ion fragmentation technique used in the analysis of the discovery set, the induced fragmentation depends on the presence or absence of a mobile proton which is required for peptide fragmentation^25^. Absence of a mobile proton results instead in selected cleavages. Wurh *et al*. reported that the efficiency of TMT^c^ formation depends on both peptide sequence and charge, with enhanced TMT^c^ formation for ions without a mobile proton^11^. This is likely the case for the selected cluster #7367, leading to the formation of TMT^c^, without peptide backbone fragmentation (Fig. 3a). Electron transfer dissociation (ETD) and EThcD, in contrast, inducing fragmentation by transferring electrons to the peptide ions, cause cleavage of the N–C_α_ bond into c- and z-ions, while leaving labile modifying groups intact, and is particularly useful for fragmenting peptides with charge states >2^26^. EThcD fragmentation induced extensive peptide backbone fragmentation of cluster #7367 that enabled its identification by de novo sequencing followed by a MS BLAST search of the identified sequence candidates.

### Biological role of pleiotrophin

PTN is a secreted growth factor and cytokine, associated with the extracellular matrix, with several functions including neural development, angiogenesis and tissue regeneration^27–29^. It is part of the neurite growth-promoting factor (NEGF) family and of importance during embryonic and early post-natal development^30^. In the adult CNS, PTN expression is limited to a few neuronal subpopulations, particularly in the hippocampus and cerebral cortex^31^, areas affected in AD. Knock-out mouse experiments suggest an involvement in learning and memory functions^30,32^.

The two most well-established PTN receptors are proteoglycan N-syndecan and chondroitin sulfate proteoglycan receptor-type protein tyrosine phosphatase ζ (PTPRZ)^33,34^. N-syndecan mediates PTN activity during neural development and PTPRZ has been associated with the ability of PTN to promote growth under normal and pathological conditions^35–38^. The affinity of PTN for these receptors is mediated through its interactions with the glucosaminoglycan part of the recpetors^33,34^. A recent study showed that the C-terminal region of PTN is essential in maintaining stable interactions with chondroitin sulphate A, the glucosaminoglycan most commonly found on PTPRZ^27^. A naturally occurring truncated form of PTN that lacks the 12 C-terminal amino acids of the full length protein, i.e., part of the 16-amino acid peptide, PTN 151–166, identified in the current study, was found to be incapable of signaling via PTPRZ^39–41^. Furthermore, a synthetic peptide with amino acid sequence corresponding to the 25 C-terminal amino acids of PTN was found to inhibit angiogenesis and PTN-induced migration and tube formation of human endothelial cells *in vitro* ^42^. Chondroitin sulfate proteoglycans have been found in postmortem brains of AD patients, in and around neurofibrillary tangles, senile plaques and dystrophic neurites^43^. Decreased CSF concentrations of PTPRZ compared to healthy controls was recently reported in a small Swedish cohort of AD patients^44^.

### Performance of PTN 151–166 as a biomarker of Alzheimer’s disease

In the present study, the peptide PTN 151–166 was identified, using an unbiased proteomic strategy, as the best candidate marker for separating AD from controls in a clinical material, and this finding was validated by targeted mass spectrometric analysis in a second cohort.

The observation that PTN 151–166 was also strongly increased in the MCI-AD group (153% in the discovery set) while the increase was less among MCI-S (35% increase) suggests that it may be an early marker of AD. Studies have shown that only about 50% of MCI patients progress to AD (MCI-AD) while the others remain in a stable MCI state (MCI-S) or develop other dementia disorders^45^. Since disease modifying drugs against AD, currently under development, are likely to be most effective if administered at an early stage of the disease, before widespread neuronal death has occurred, it is important to identify MCI-AD subjects as early as possible. Our results thus suggest that PTN 151–166 may be useful for this task.

In the validation set, which besides AD patients, also included patients with PD and PSP, PTN 151–166 was significantly increased in AD but not in the other disease groups, indicating that it may be a specific marker of AD (Fig. 4a,e). While PTN 151–166 exhibited lower performance for separating AD from controls compared to the core biomarkers in the validation set (Fig. 4f), it should be kept in mind that this difference may to a large degree reflect differences in the performance of the analytical methods used rather than in the performance of the respective biomarkers; the TMT10-plex method used for quantifying PTN 151–166 is designed for global peptide quantification in explorative studies while Aβ42, T-tau, and P-tau were measured using highly optimized ELISA assays that can be expected to have lower variation. Further studies will be required to assess the biomarker performance of PTN 151–166 and detemine whether it provides information of value in addition to the core AD biomarkers.

At present, there are no clinical studies on CSF pleotrophin in AD. The finding that PTN 151–166 concentrations in CSF are increased in AD patients compared to both cognitively normal control individuals and patients with other neurodegenerative disorders motivates further study of extracellular matrix changes in relation to AD neuropathology. Our observation of decreased PTN 151–166 in MCI-AD patients compared to AD patients, and its correlation with the established biomarkers, t-tau, P-tau and beta amyloid, suggests an association with disease progress that should be further explored.

## Methods

### Samples

The discovery sample set included in this study were taken from the memory clinic based Amsterdam Dementia Cohort^46^, and selected based on the availability of baseline CSF, which was collected according to international consensus guidelines^47^. Forty patients with a diagnosis of probable AD were matched for age and sex to 40 controls with subjective cognitive decline and 40 patients with MCI. All subjects in the dataset were used in method validation. However, when comparing subject groups in the search for potential new biomarkers in this dataset we used the IWG-2 criteria^10^ to select subjects with a clear AD biomarker profile, and excluded seven subjects that did not meet the criteria. We used the following cutoffs for IWG-2 classification: Aβ42 ≤650 pg/mL, t-tau ≥375 pg/mL, P-tau >52 pg/mL for patients <60 years, and P-tau >80 for patients ≥60 years. To fulfill the IWG-2 criteria, patients should test positive for Aβ42 and positive for t-tau or P-tau or both. Further, in the MCI group, 4 subjects who were diagnosed with other dementia disorders than AD at follow up were also excluded. The MCI patients were sub-classified into an MCI-AD group, for MCI patients that progressed into AD at follow-up, and an MCI-S group, for patients who had no clinical progression of their MCI status at follow-up. All subjects gave written informed consent for the use of their clinical data for research purposes, and use of the samples for research was approved by the local Medical Ethics Committee at VU University Medical Center, Amsterdam. The CSF samples were collected in polypropylene tubes, centrifuged (1800 * g, 10 minutes, +4 °C) and the collected supernatant was stored at −80 °C pending biochemical analysis. All methods were performed in accordance with the guidelines and regulations of the Sahlgrenska Academy at the University of Gothenburg.

The biomarker validation set used in this study consisted of 15 AD patients, 15 PD patients, 15 PSP patients and 15 healthy controls, recruited at the Skåne University Hospital, Sweden, as part of the prospective Swedish BioFINDER study (www.biofinder.se). All study subjects were examined for neurological and cognitive deficits by experienced medical doctors and nurses. The controls were healthy elderly cases without any subjective or objective cognitive decline. The patients with AD fulfilled the criteria for probable AD according to the recommendations from the National Institute on Aging-Alzheimer’s Association workgroups on diagnostic guidelines for Alzheimer’s disease^48^, the PD patients met the NINDS Diagnostic Criteria for PD^49^, and the patients with PSP met the criteria according to the report of the National Institute of Neurological Disorders and Stroke–Society for Progressive Supranuclear Palsy International Workshop^50^.

Aβ42, t-tau, and P-tau were measured using ELISA assays (Innotest, Belgium) according to the manufacturer’s protocol.

### TMT labelling of clinical CSF samples

The CSF samples (100 µl) were mixed with 50 µl 8 M guanidine hydrochloride solution (Sigma-Aldrich) and 15.6 µl 1 M triethylammonium bicarbonate buffer (TEAB) (Sigma-Aldrich). Cysteine disulfides were reduced by addition of 4.2 µl 200 mM tris(2-carboxyethyl)phosphine (TCEP) (Thermo Scientific) and incubation at 55 °C for 1 hour after which the samples were allowed to cool to room temperature. To alkylate cysteines, 4.3 µl 400 mM iodoacetamide (Sigma) were added, followed by incubation at room temperature in the dark for 30 min. TMT 10-plex reagents (Thermo Scientific) were dissolved in acetonitrile (ACN) to a concentration of 19.5 mg/mL and 19.5 µl of the reagent solution were added to each sample, after which they were incubated for 1 hour at room temperature. The TMT labeling reaction was then quenched by adding 9.7 µl of 5% (v/v) hydroxylamine (Sigma-Aldrich) to the samples, after which they were pooled into TMT sets.

The study samples were randomly distributed over the TMT sets, the discovery study (120 samples) comprising 14 TMT sets and the validation study (60 samples) comprising seven. A reference sample, prepared by pooling aliquots of all CSF samples in the respective study was labeled with TMT-131 and used for inter-TMT set data normalization.

### Isolation of endogenous CSF peptides

The endogenous peptide fraction was isolated from the TMT-labeled CSF samples using molecular weight cut-off (MWCO) filters (Amicon Ultra-15 Centrifugal Filter Units 30 kDa [UFC903024], Merck Millipore), operated by centrifugation at 2,500 × g at room temperature, as previously described^24^. The filter devices were pre-washed with 1 ml of 100 mM TEAB. The TMT-labeled CSF samples were loaded and the peptide fraction was collected in the flow-through. An additional 200 µl 100 mM TEAB were loaded and spun through to increase peptide recovery. The peptide fraction was diluted with water to decrease the ACN concentration to < 5% and the samples were acidified by addition of 10% (v/v) trifluoroacetic acid (TFA) to pH < 3, and subsequently desalted using SEP-PAK C_18_ cartridges (1cc, 100 mg, Waters), dried by vacuum centrifugation and stored at −80 °C pending analysis.

### LC-ESI MS

The samples belonging to the discovery set were reconstituted in 6 µl 2% acetonitrile, 0.1% TFA (Loading Buffer). Aliquots of 5 µl were loaded on a nano-LC (Ultimate 3000, Thermo Scientific) equipped with a C_18_ trap column (PepMap Acclaim 75 µm *20 mm, Thermo Scientific), and a C_18_ separation column (PepMap Acclaim 75 µm * 500 mm, Thermo Scientific), coupled to a Q-Exactive electrospray ionization mass spectrometer (Thermo Scientific), fitted with a FlexiSpray ion source. The loading buffer was 2% acetonitrile, 0.05% TFA; Buffer A was 0.1% formic acid; and Buffer B was 84% acetonitrile, 0.1% formic acid. The following gradient was used: t = 0 min, B = 3%; 140 min, B = 30%; 160 min, B = 45%; 165 min, B = 80%. The mass spectrometer was operated in the positive ion mode. Data-dependent acquisition was used, acquiring one full MS scan (R 140k, AGC target 3e6, max IT 250 ms, scan range 400 to 1600 m/z) and up to 10 consecutive HCD MS/MS scans (R = 70k, AGC target = 1e6, max IT = 250 ms, isolation window 1.2 m/z, NCE 32.0, charge exclusion: unassigned, >6). Samples were analyzed in quadruplicate.

The validation sample set was analyzed on a Tribrid Fusion MS (Thermo), also fitted with a FlexiSpray ion source. The LC model, configuration and method were the same as above. One full scan MS spectrum was recorded (R = 140k, AGC target = 3e6, max IT = 250 ms) followed by targeted analysis of *m/z* 794.115 (+5) ion, using HCD fragmentation (R = 70k, AGC target = 1e6, max IT = 250 ms, isolation window = 1.2 m/z, NCE = 32.0, charge exclusion: unassigned, >6).

Sequencing of the identified biomarker candidate was performed on the Fusion mass spectrometer, using EThcD using the default calibrated charge dependent electron transfer dissociation parameters (other parameters: 60 K, max IT 100 ms, AGC target 5e4).

### Peptide identification

Peptide identification was performed using Proteome Discoverer 1.4 (Thermo Scientific) using Mascot (MatrixScience) for database searching, and PEAKS Studio (BSI). The search settings were: precursor ∆m tolerance = 10 ppm, fragment ∆m tolerance = 20 milli mass units, missed cleavages = 2, fixed modifications = carbamidomethylation, variable modifications = oxidation of methionine), searching the human subset of the UniProtKB Swiss-Prot database (release 13–10) (www.uniprot.org). Percolator (MatrixScience) was used for scoring peptide specific matches, and 1% false discovery rate (FDR) was set as threshold for identification.

Automatic *de novo* sequencing was performed by the auto-*de novo* function in PEAKS Studio. Peptide sequence tags derived from spectra were searched using MS Blast^7^ (http://genetics.bwh.harvard.edu/msblast/). Matching candidate amino acid sequences from BLAST searches to mass spectrometric data was assisted by use of the software programs GPMAW (Lighthouse Data) and mMass^51^.

### Spectral clustering and quantification

Spectral clustering was performed using MS-Cluster v2^8^, an open source software for TMT dataset clustering. MS-Cluster uses a hierarchical clustering algorithm similar to the Pep-Miner algorithm^52^, but is optimized for analysis of large numbers of mass spectra. The algorithm clusters spectra in the TMT dataset by similarity using the normalized dot-product, which has shown good results in several studies^52–54^. The raw MS data was converted to peak lists in the mgf format using the software Proteome Discoverer 1.4 (Thermo Scientific). A Δ*m/z* window of 0.005 was used for TMT reporter ion detection. Prior to clustering, the TMT reporter ions were temporarily deleted from the data, as not to affect clustering. For the purposes of this study, the clustering algorithm was tuned to minimize the risk of generating clusters containing fragment spectra from divergent peptides, at the calculated cost of a higher probability of rendering cases of split clusters, where the same peptide spectra is spread out over several clusters. This was considered preferable as divergent peptide clusters would result in a higher probability of false positives in the search of new possible biomarkers of disease. The following parameters were used for clustering: sqs 0.5, mixture probability 0.025 and fragment tolerance 0.005.

Within each TMT set, the reporter ion intensities of each TMT channel were normalized to the median intensity of the reporter ion intensities for that channel over all spectra to reduce the influence of experimental variation that affect individual samples, such as pipetting error. For each LC-MS data set, the intensity ratio of each TMT reporter (126–130 C) to the TMT-131 reporter (representing the common reference sample) was calculated to reduce the influence of run-to-run variations.

### Statistics

The performance of each cluster in the discovery set as a distinguishing factor between the controls and the AD patients was calculated using ROC analysis in R (ver 3.1.1)^55^. Kruskall-Wallis test with Dunn’s multiple comparisons test was performed using GraphPad Prism version 6.00 for Windows (GraphPad Software, La Jolla California USA, www.graphpad.com). Mann-Whitney test were used to calculate the significance p-values shown in Figs 2 and 4. Correlations and group differences were calculated using Spearman’s Rank-Order correlations and Mann-Whitney U analysis in SPSS statistics v.22 (IBM, New York).

## Electronic supplementary material

Supplementary tables

Supplementary Dataset
