# Supplementary material for: A novel quantification-driven proteomic strategy identifies an endogenous peptide of pleiotrophin as a new biomarker of Alzheimer’s disease
Source: Sci Rep. 2017 Oct 17;7:13333. doi: 10.1038/s41598-017-13831-0 (PMC5645330; doi:10.1038/s41598-017-13831-0)
Supplement: Supplementary file 1 — Supplementary tables [file 41598_2017_13831_MOESM1_ESM.doc]

**A novel quantification-driven proteomic strategy identifies an endogenous peptide of pleiotrophin as a new biomarker of Alzheimer’s disease**

Tobias Skillbäck, Niklas Mattsson, Karl Hansson, Ekaterina Mirgorodskaya, Rahil Dahlén, Wiesje van der Flier, Philip Scheltens, Floor Duits, Oskar Hansson, Charlotte Teunissen, Kaj Blennnow, Henrik Zetterberg, Johan Gobom

# Supplementary Tables

### Supplementary Table S1. Demographics of study population in the Discovery sample set

| **Diagnosis** | | **C** | **MCI-S** | **MCI-AD** | **MCI-OD** | **AD (IWG-2)** | **Prob. AD** |
| --- | --- | --- | --- | --- | --- | --- | --- |
| **Sex** | F | 19 | 11 | 7 | 1 | 17 | 2 |
|  | M | 21 | 12 | 7 | 2 | 16 | 5 |
| **Age (yrs)** | Mean  (SD) | 64.5 (8.2) | 63.7 (7.4) | 65.6 (9.0) | 64.4 (11.7) | 65.5 (8.2) | 61.11 (6.6) |
|  | Median (IQR) | 63.2 (57.9-71.2) | 63.9 (57.9-69.6) | 67.7 (57.7-71.5) | 61.5 (54.4-77.3) | 66.7 (57.7-71.2) | 59.6 (54.3-62.7) |
| **MMSE (points)** | Mean (SD) | 28 (2) | 27 (2) | 26 (3) | 26 (3) | 21 (4) | 23 (4) |
|  | Median (IQR) | 29 (28-29) | 27 (25-28) | 26 (24-28) | 24 (24-29) | 22 (19-24) | 24 (19-26) |

C = controls; MCI-S = stable MCI; MCI-AD = MCI patients that progressed to AD; MCI-OD = MCI patients that progressed to other diseases; AD (IWG-2) = Patients that fulfilled the IWG-2 criteria for AD at follow-up; Prob. AD = Patients that fulfilled clinical but not IWG2 criteria for AD at follow-up

### Supplementary Table S2. Peptide fragments ion matching PTN 151-166

| **Meas. m/z** | **Calc. m/z** | **δ (Da)** | **δ (ppm)** | **Int.** | **Rel. Int. (%)** | **Annotation** |
| --- | --- | --- | --- | --- | --- | --- |
| 318.2325 | 318.2339 | -0.0014 | -4.39928 | 1349 | 2.72 | c'1 (1+) |
| 447.2744 | 447.2764 | -0.002 | -4.47151 | 5049 | 10.19 | c'2 (1+) |
| 534.3056 | 534.3085 | -0.0029 | -5.42758 | 1816 | 3.66 | c'3 (1+) |
| 624.9134 | 624.9158 | -0.0024 | -3.84052 | 546 | 1.1 | c'5 (2+) |
| 749.4008 | 749.4059 | -0.0051 | -6.80539 | 100 | 0.2 | z''4 (1+) |
| 760.972 | 760.9752 | -0.0032 | -4.20513 | 510 | 1.03 | z''7 (2+) |
| 803.5414 | 803.5447 | -0.0033 | -4.1068 | 1312 | 2.65 | c'6 (2+) |
| 817.2163 | 817.2183 | -0.002 | -2.44733 | 219 | 0.44 | c'9 (3+) |
| 836.222 | 836.2255 | -0.0035 | -4.18547 | 525 | 1.06 | c'10 (3+) |
| 891.5617 | 891.5664 | -0.0047 | -5.27162 | 3271 | 6.6 | c'4 (1+) |
| 907.9184 | 907.9246 | -0.0062 | -6.82876 | 273 | 0.55 | z''11 (3+) |
| 935.3504 | 955.3115 | -19.9611 | -20894.9 | 4739 | 9.56 | c'11 (3+) |
| 959.6357 | 959.6397 | -0.004 | -4.16823 | 7157 | 14.44 | c'15 (4+) |
| 982.1697 | 982.1737 | -0.004 | -4.0726 | 844 | 1.7 | c'7 (2+) |
| 1004.118 | 1004.125 | -0.0074 | -7.3696 | 155 | 0.31 | z''9 (2+) |
| 1027.0041 | 1027.011 | -0.0065 | -6.32905 | 376 | 0.76 | z''12 (3+) |
| 1074.3919 | 1074.398 | -0.0056 | -5.21222 | 5791 | 11.68 | c'12 (3+) |
| 1106.663 | 1106.664 | -0.0008 | -0.72289 | 1275 | 2.57 | z''5 (1+) |
| 1117.0779 | 1117.084 | -0.0057 | -5.10257 | 8681 | 17.51 | c'13 (3+) |
| 1146.089 | 1146.097 | -0.0076 | -6.6312 | 306 | 0.62 | z''13 (3+) |
| 1160.0915 | 1160.098 | -0.0063 | -5.43058 | 7404 | 14.94 | c'14 (3+) |
| 1175.1028 | 1175.107 | -0.0044 | -3.74434 | 1902 | 3.84 | z''14 (3+) |
| 1182.7444 | 1182.754 | -0.01 | -8.45484 | 518 | 1.05 | z''10 (2+) |
| 1218.1137 | 1218.121 | -0.0077 | -6.32121 | 4042 | 8.16 | z''15 (3+) |
| 1225.3182 | 1225.324 | -0.0057 | -4.65183 | 4465 | 9.01 | c'9 (2+) |
| 1253.8279 | 1253.835 | -0.0068 | -5.42336 | 5729 | 11.56 | c'10 (2+) |
| 1279.1781 | 1279.184 | -0.0057 | -4.45597 | 705 | 1.42 | c'15 (3+) |
| 1361.3759 | 1361.383 | -0.0074 | -5.43565 | 1080 | 2.18 | z''11 (2+) |
| 1432.4561 | 1432.464 | -0.0075 | -5.23574 | 1579 | 3.19 | c'11 (2+) |
| 1463.9138 | 1463.922 | -0.0079 | -5.39646 | 517 | 1.04 | z''6 (1+) |
| 1520.9337 | 1520.943 | -0.0094 | -6.18038 | 829 | 1.67 | z''7 (1+) |
| 1540.0029 | 1540.012 | -0.0094 | -6.10385 | 405 | 0.82 | z''12 (2+) |
| 1606.0707 | 1606.082 | -0.0115 | -7.16028 | 223 | 0.45 | c'6 (1+) |
| 1611.0805 | 1611.093 | -0.012 | -7.44836 | 565 | 1.14 | c'12 (2+) |
| 1675.112 | 1675.122 | -0.0098 | -5.85032 | 1402 | 2.83 | c'13 (2+) |
| 1718.6295 | 1718.641 | -0.0117 | -6.8077 | 334 | 0.67 | z''13 (2+) |
| 1739.6335 | 1739.643 | -0.0096 | -5.51837 | 1671 | 3.37 | c'14 (2+) |
| 1762.1508 | 1762.157 | -0.0064 | -3.63191 | 759 | 1.53 | z''14 (2+) |
| 1826.6686 | 1826.679 | -0.0099 | -5.41967 | 2280 | 4.6 | z''15 (2+) |

Meas. m/z = measured monoisotopic m/z; Calc.m/z = calculated monoisotopic m/z; δ (Da) = m/z deviation in Dalton; δ (ppm) = m/z deviation in ppm; Rel. Int. (%) = peak intensity relative to base peak; Annotation = fragment ion and charge.

### Supplementary Table S3. Demographics of study population in the Validation sample set

| **Diagnosis** | | **C** | **AD** | **PD** | **PSP** |
| --- | --- | --- | --- | --- | --- |
| **Sex** | F | 7 | 7 | 7 | 7 |
|  | M | 8 | 8 | 8 | 8 |
| **Age (yrs)** | Mean (SD) | 70.0 (5.2) | 70.7 (6.0) | 69.3 (6.1) | 69.9 (7.0) |
|  | Median (IQR) | 69.0 (65.0-73.0) | 71.0 (66.0-74.0) | 69.3 (64.0-74.0) | 70.0 63.0-73.0) |
| **MMSE (points)** |  | 29 (1) | 18 (4) | 28 (2) | 25 (5) |
|  |  | 29 (29-30) | 19 (18-21) | 28 (27-29) | 27 (22-28) |

C = controls; AD = Alzheimer’s disease; PD = Parkinson’s Disease; PSP = progressive supranuclear palsy.

**Supplementary Table S4. Validation of pleiotrophin 151-166 as a biomarker of Alzheimer’s disease and comparison to the core biomarkers**

| **Biomarker** | **Comparison** | **Rel. diff.** | **Adjusted P value** |
| --- | --- | --- | --- |
| **PTN 151-166** | AD vs. C | 35% | 0.0093 |
|  | PD vs. C | -2% | >0.9999 |
|  | PSP vs. C | 12% | 0.5955 |
| **Aβ42** | AD vs. C | -57% | <0.0001 |
|  | PD vs. C | -31% | 0.0356* |
|  | PSP vs. C | -45% | 0.0029 |
| **T-tau** | AD vs. C | 131% | 0.0002 |
|  | PD vs. C | -22% | >0.9999 |
|  | PSP vs. C | 6% | >0.9999 |
| **P-tau** | AD vs. C | 66% | 0.0022 |
|  | PD vs. C | -20% | >0.9999 |
|  | PSP vs. C | -14% | >0.9999 |

The abundances of PTN 151-166, Aβ42, T-tau, and P-tau in AD, PD and PSP patients were compared to healthy controls (C). Relative diff. = median relative difference between the groups. Adjusted P values were calculated using Kruskall-Wallis test with Dunn’s multiple comparisons test. Significant results (p<0.01) are highlighted.

**Supplementary Table S5. Correlations of PTN 151-166 with the core AD biomarkers in the discovery set**

| **Diagnosis** | | | **PTN 151-166** | Aβ42 | T-tau | P-tau |
| --- | --- | --- | --- | --- | --- | --- |
| **C** | **PTN 151-166** | Corr. coeff. | 1.000 | -.262 | .170 | -.121 |
|  |  | Sig. (2-tailed) |  | .107 | .301 | .462 |
|  |  | N | 39 | 39 | 39 | 39 |
|  | Aβ42 | Corr. coeff. | -.262 | 1.000 | .251 | .314 |
|  |  | Sig. (2-tailed) | .107 |  | .124 | .051 |
|  |  | N | 39 | 39 | 39 | 39 |
|  | T-tau | Corr. coeff. | .170 | .251 | 1.000 | ,599^**^ |
|  |  | Sig. (2-tailed) | .301 | .124 |  | .000 |
|  |  | N | 39 | 39 | 39 | 39 |
|  | P-tau | Corr. coeff. | -.121 | .314 | ,599^**^ | 1.000 |
|  |  | Sig. (2-tailed) | .462 | .051 | .000 |  |
|  |  | N | 39 | 39 | 39 | 39 |
| **AD** | **PTN 151-166** | Corr. coeff. | 1.000 | -,505^**^ | ,570^**^ | ,493^**^ |
|  |  | Sig. (2-tailed) |  | .003 | .001 | .004 |
|  |  | N | 33 | 33 | 33 | 33 |
|  | Aβ42 | Corr. coeff. | -,505^**^ | 1.000 | -.239 | -.293 |
|  |  | Sig. (2-tailed) | .003 |  | .180 | .098 |
|  |  | N | 33 | 33 | 33 | 33 |
|  | Ttau | Corr. coeff. | ,570^**^ | -.239 | 1.000 | ,822^**^ |
|  |  | Sig. (2-tailed) | .001 | .180 |  | .000 |
|  |  | N | 33 | 33 | 33 | 33 |
|  | Ptau | Corr. coeff. | ,493^**^ | -.293 | ,822^**^ | 1.000 |
|  |  | Sig. (2-tailed) | .004 | .098 | .000 |  |
|  |  | N | 33 | 33 | 33 | 33 |
| **MCI-AD** | **PTN 151-166** | Corr. coeff. | 1.000 | .020 | ,719^**^ | .442 |
|  |  | Sig. (2-tailed) |  | .946 | .004 | .114 |
|  |  | N | 14 | 14 | 14 | 14 |
|  | Aβ42 | Corr. coeff. | .020 | 1.000 | .077 | -.279 |
|  |  | Sig. (2-tailed) | .946 |  | .794 | .334 |
|  |  | N | 14 | 14 | 14 | 14 |
|  | Ttau | Corr. coeff. | ,719^**^ | .077 | 1.000 | ,714^**^ |
|  |  | Sig. (2-tailed) | .004 | .794 |  | .004 |
|  |  | N | 14 | 14 | 14 | 14 |
|  | Ptau | Corr. coeff. | .442 | -.279 | ,714^**^ | 1.000 |
|  |  | Sig. (2-tailed) | .114 | .334 | .004 |  |
|  |  | N | 14 | 14 | 14 | 14 |
| **MCI-S** | **PTN 151-166** | Correlation Coefficient | 1.000 | -,715^**^ | .253 | .136 |
|  |  | Sig. (2-tailed) |  | .000 | .283 | .567 |
|  |  | N | 20 | 20 | 20 | 20 |
|  | Aβ42 | Corr. coeff. | -,715^**^ | 1.000 | -.021 | .015 |
|  |  | Sig. (2-tailed) | .000 |  | .930 | .950 |
|  |  | N | 20 | 20 | 20 | 20 |
|  | Ttau | Corr. coeff. | .253 | -.021 | 1.000 | ,928^**^ |
|  |  | Sig. (2-tailed) | .283 | .930 |  | .000 |
|  |  | N | 20 | 20 | 20 | 20 |
|  | Ptau | Corr. coeff. | .136 | .015 | ,928^**^ | 1.000 |
|  |  | Sig. (2-tailed) | .567 | .950 | .000 |  |
|  |  | N | 20 | 20 | 20 | 20 |

** Correlation is significant at the 0.01 level (2-tailed). * Correlation is significant at the 0.05 level (2-tailed).

Spearman’s rank order correlation of CSF biomarkers and demographic data. PTN 151-166 = pleiotrophin 151-166 relative abundance; Aβ42 = β-amyloid 1-42 concentration; P-tau = phospho-tau concentration. Significant (p<0.05) correlations are highlighted.

**Supplementary Table S6. Correlations of PTN 151-166 with the core AD biomarkers in the validation set**

| **Diagnosis** |  |  | **PTN 151-166** | Aβ42 | tau | Ptau |
| --- | --- | --- | --- | --- | --- | --- |
| **C** | **PTN 151-166** | Corr. coeff. | 1.00 | 0.46 | ,629^*^ | ,656^*^ |
|  |  | Sig. (2-tailed) |  | 0.13 | 0.03 | 0.02 |
|  |  | N | 12.00 | 12.00 | 12.00 | 12.00 |
|  | Aβ42 | Corr. coeff. | 0.46 | 1.00 | -0.06 | 0.01 |
|  |  | Sig. (2-tailed) | 0.13 |  | 0.86 | 0.97 |
|  |  | N | 12.00 | 12.00 | 12.00 | 12.00 |
|  | tau | Corr. coeff. | ,629^*^ | -0.06 | 1.00 | ,986^**^ |
|  |  | Sig. (2-tailed) | 0.03 | 0.86 |  | 0.00 |
|  |  | N | 12.00 | 12.00 | 12.00 | 12.00 |
|  | Ptau | Corr. coeff. | ,656^*^ | 0.01 | ,986^**^ | 1.00 |
|  |  | Sig. (2-tailed) | 0.02 | 0.97 | 0.00 |  |
|  |  | N | 12.00 | 12.00 | 12.00 | 12.00 |
| **AD** | **PTN 151-166** | Corr. coeff. | 1.00 | -0.16 | 0.37 | ,514^*^ |
|  |  | Sig. (2-tailed) |  | 0.56 | 0.17 | 0.05 |
|  |  | N | 15.00 | 15.00 | 15.00 | 15.00 |
|  | Aβ42 | Corr. coeff. | -0.16 | 1.00 | -0.03 | -0.02 |
|  |  | Sig. (2-tailed) | 0.56 |  | 0.93 | 0.95 |
|  |  | N | 15.00 | 15.00 | 15.00 | 15.00 |
|  | tau | Corr. coeff. | 0.37 | -0.03 | 1.00 | ,936^**^ |
|  |  | Sig. (2-tailed) | 0.17 | 0.93 |  | 0.00 |
|  |  | N | 15.00 | 15.00 | 15.00 | 15.00 |
|  | Ptau | Corr. coeff. | ,514^*^ | -0.02 | ,936^**^ | 1.00 |
|  |  | Sig. (2-tailed) | 0.05 | 0.95 | 0.00 |  |
|  |  | N | 15.00 | 15.00 | 15.00 | 15.00 |
| **PD** | **PTN 151-166** | Corr. coeff. | 1.00 | 0.45 | ,515^*^ | ,564^*^ |
|  |  | Sig. (2-tailed) |  | 0.09 | 0.05 | 0.03 |
|  |  | N | 15.00 | 15.00 | 15.00 | 15.00 |
|  | Aβ42 | Corr. coeff. | 0.45 | 1.00 | 0.27 | 0.40 |
|  |  | Sig. (2-tailed) | 0.09 |  | 0.33 | 0.14 |
|  |  | N | 15.00 | 15.00 | 15.00 | 15.00 |
|  | tau | Corr. coeff. | ,515^*^ | 0.27 | 1.00 | ,831^**^ |
|  |  | Sig. (2-tailed) | 0.05 | 0.33 |  | 0.00 |
|  |  | N | 15.00 | 15.00 | 15.00 | 15.00 |
|  | Ptau | Corr. coeff. | ,564^*^ | 0.40 | ,831^**^ | 1.00 |
|  |  | Sig. (2-tailed) | 0.03 | 0.14 | 0.00 |  |
|  |  | N | 15.00 | 15.00 | 15.00 | 15.00 |
| **PSP** | **PTN 151-166** | Corr. coeff. | 1.00 | 0.15 | 0.36 | 0.58 |
|  |  | Sig. (2-tailed) |  | 0.65 | 0.27 | 0.06 |
|  |  | N | 11.00 | 11.00 | 11.00 | 11.00 |
|  | Aβ42 | Corr. coeff. | 0.15 | 1.00 | -0.01 | 0.16 |
|  |  | Sig. (2-tailed) | 0.65 |  | 0.98 | 0.63 |
|  |  | N | 11.00 | 11.00 | 11.00 | 11.00 |
|  | tau | Corr. coeff. | 0.36 | -0.01 | 1.00 | ,738^**^ |
|  |  | Sig. (2-tailed) | 0.27 | 0.98 |  | 0.01 |
|  |  | N | 11.00 | 11.00 | 11.00 | 11.00 |
|  | Ptau | Corr. coeff. | 0.58 | 0.16 | ,738^**^ | 1.00 |
|  |  | Sig. (2-tailed) | 0.06 | 0.63 | 0.01 |  |
|  |  | N | 11.00 | 11.00 | 11.00 | 11.00 |

** Correlation is significant at the 0.01 level (2-tailed). * Correlation is significant at the 0.05 level (2-tailed).

Spearman’s rank order correlation of CSF biomarkers and demographic data. PTN 151-166 = pleiotrophin 151-166 relative abundance; Aβ42 = β-amyloid 1-42 concentration; P-tau = phospho-tau concentration. Significant (p<0.05) correlations are highlighted.
